# Preservation of Interference Effects in Working Memory After Orbitofrontal Damage

**DOI:** 10.3389/fnhum.2019.00445

**Published:** 2020-01-08

**Authors:** Anaïs Llorens, Ingrid Funderud, Alejandro O. Blenkmann, James Lubell, Maja Foldal, Sabine Leske, Rene Huster, Torstein R. Meling, Robert T. Knight, Anne-Kristin Solbakk, Tor Endestad

**Affiliations:** ^1^Department of Neurosurgery, Oslo University Hospital-Rikshospitalet, Oslo, Norway; ^2^Department of Psychology, University of Oslo, Oslo, Norway; ^3^Department of Psychology, Helen Wills Neuroscience Institute, University of California, Berkeley, Berkeley, CA, United States; ^4^RITMO Centre for Interdisciplinary Studies in Rhythm, Time and Motion, University of Oslo, Oslo, Norway; ^5^Faculty of Medicine, University of Oslo, Oslo, Norway; ^6^Service de Neurochirurgie, Hôpitaux Universitaires de Genève, Genève, Switzerland; ^7^Department of Neuropsychology, Helgeland Hospital, Mosjøen, Norway

**Keywords:** orbitofrontal cortex, recent probes task, working memory, recency, event-related potentials

## Abstract

Orbitofrontal cortex (OFC) is implicated in multiple cognitive processes, including inhibitory control, context memory, recency judgment, and choice behavior. Despite an emerging understanding of the role of OFC in memory and executive control, its necessity for core working memory (WM) operations remains undefined. Here, we assessed the impact of OFC damage on interference effects in WM using a Recent Probes task based on the Sternberg item-recognition task (1966). Subjects were asked to memorize a set of letters and then indicate whether a probe letter was presented in a particular set. Four conditions were created according to the forthcoming response (“yes”/“no”) and the recency of the probe (presented in the previous trial set or not). We compared behavioral and electroencephalography (EEG) responses between healthy subjects (*n* = 14) and patients with bilateral OFC damage (*n* = 14). Both groups had the same recency pattern of slower reaction time (RT) when the probe was presented in the previous trial but not in the current one, reflecting the proactive interference (PI). The within-group electrophysiological results showed no condition difference during letter encoding and maintenance. In contrast, event-related potentials (ERPs) to probes showed distinct within-group condition effects, and condition by group effects. The response and recency effects for controls occurred within the same time window (300–500 ms after probe onset) and were observed in two distinct spatial groups including right centro-posterior and left frontal electrodes. Both clusters showed ERP differences elicited by the response effect, and one cluster was also sensitive to the recency manipulation. Condition differences for the OFC group involved two different clusters, encompassing only left hemisphere electrodes and occurring during two consecutive time windows (345–463 ms and 565–710 ms). Both clusters were sensitive to the response effect, but no recency effect was found despite the behavioral recency effect. Although the groups had different electrophysiological responses, the maintenance of letters in WM, the evaluation of the context of the probe, and the decision to accept or reject a probed letter were preserved in OFC patients. The results suggest that neural reorganization may contribute to intact recency judgment and response after OFC damage.

## Introduction

The orbitofrontal cortex (OFC)^1^ is involved in several high-level cognitive functions such as goal-directed attention (Luu et al., 2000; Badre and Wagner, 2004; Ullsperger and Cramon, 2004; Walton et al., 2004; Hebscher and Gilboa, 2016), inhibitory control (Godefroy et al., 1999; Picton et al., 2007), and decision-making (Bechara et al., 1998, 2000; Ernst et al., 2002; Hebscher and Gilboa, 2016). Moreover, several studies have highlighted its role in working memory (WM). Although damage confined to the OFC does not typically lead to deficient WM performance when assessed with standard neuropsychological tests, it has been reported that OFC lesions can impair performance on tasks involving the coordination of WM maintenance, manipulation, and monitoring processes such as in N-back tasks (Wager and Smith, 2003; Owen et al., 2005; Barbey et al., 2011, 2013) and Delayed match-to-sample tasks (Meunier, 1997; Schon et al., 2008). These results are in line with functional magnetic resonance imaging (fMRI) studies in healthy subjects where OFC was found to be involved in WM maintenance, interference control, and inhibition during delayed-response tasks (D’Esposito et al., 2000). It has also been reported that OFC damage leads to difficulty learning from previous errors (Bechara et al., 2000; Stuss et al., 2000) and deficits in context memory (Janowsky et al., 1989; Duarte et al., 2010). OFC has also been implicated in recency memory (Shimamura et al., 1990; Incisa della Rocchetta and Milner, 1993; for human studies, and Barker et al., 2007; Devito and Eichenbaum, 2011 for rodent studies).

Given the role of the OFC in context encoding, familiarity, and recency judgments, as well as decision-making and confidence during memory retrieval, we aimed to study the impact of damage in this brain region on the ability to perform a WM task involving such cognitive processes. We used the Recent Probes task which is based on the item-recognition task of Sternberg (1966). Subjects were asked to memorize a set of items (target set) and, when prompted, to indicate whether a given item (probe) was presented in the current set (Sternberg, 1966). This task requires the encoding and maintenance of verbal material in WM, as well as the evaluation of the recency and familiarity of the probe with the current set of items. This is particularly relevant in order to correctly reject any lures, i.e., probes presented in the previous but not the current trial, leading to item-specific proactive interference (PI; Monsell, 1978). Moreover, this task entails decision-making regarding the presence or not of the probe in the current set of items (Badre and Wagner, 2004; Kan and Thompson-Schill, 2004; Jonides and Nee, 2006).

The brain network engaged in the Recent Probes task includes several subregions of the frontal lobe with major involvement of the left ventrolateral prefrontal cortex (VLPFC) and the dorsolateral prefrontal cortex (DLPFC). The VLPFC prevents irrelevant information from becoming active, i.e., PI resolution (Postle et al., 2004; fMRI studies: Jonides et al., 1998; D’Esposito et al., 1999a; Mecklinger et al., 2003; Badre and Wagner, 2004; Jonides and Nee, 2006; TMS study: Feredoes et al., 2006; lesion study: Thompson-Schill et al., 2002). The DLPFC is involved in monitoring and manipulating cognitive representations in WM (Wager et al., 2005; Burgess and Braver, 2010; Barbey et al., 2013). Interestingly, Badre and Wagner (2005) showed that the activation of the rostral part of OFC (BA10) was negatively correlated with PI for probes recently presented in a Recent-Probes task (see also Mecklinger et al., 2003). Despite other regions’ involvement in PI, the authors concluded that OFC was partly responsible for monitoring the familiarity of the probe (Elliott et al., 2000; Simons and Spiers, 2003), and for encoding the context that subsequently guides memory retrieval (Stuss et al., 1982; Farovik et al., 2015). Similarly, Nee et al. (2007) reported the involvement of the left OFC in the resolution of PI in a Recent Probes task and in a Directed-Forgetting task. Only a few studies have examined the encoding and maintenance phases of the Recent Probes task. They reported that neither of the two phases seem to directly impact the PI resolution (D’Esposito et al., 1999b; Badre and Wagner, 2005; Braver et al., 2007).

Several electrophysiological studies using event-related potentials (ERPs) have attempted to characterize the temporal dynamics involved in the resolution of PI during the Recent Probes task. However, the debate about which ERP components reflect the recency effect remains unsettled. Some studies have reported the presence of a frontal negativity that peaks between 250 and 350 ms (N2) after the probe presentation and is related to the degree of mismatch and response-conflict induced by PI (Du et al., 2008; Folstein and Van Petten, 2008), whereas others have reported a later negativity at frontal sites peaking around 420 ms (Yi and Friedman, 2014; see also Tays et al., 2008, 2009). Finally, Zhang et al. (2010) reported an N2 and a P3, which were both only modulated by the decision whether the item was present or not in the current set, and a late positive component (LPC) modulated by the PI resolution. All these studies involved healthy participants.

In the current study, we aimed to elucidate the role of the OFC in a Recent Probes task by comparing the behavioral performance and the electrophysiological markers of healthy controls and patients with focal OFC damage. We focused on the probe period to examine whether the OFC lesion impacts the resolution of PI in WM. The influence of OFC lesion on the letter encoding or maintenance phases was also evaluated. Based on the literature, we hypothesized that the patients’ behavioral performance would be impacted by altered familiarity and recency judgment processes, manifested by prolonged reaction times (RTs), stronger PI, and increased error rates. We aimed to extend current knowledge about the electrophysiological modulations elicited by the probe presentation in a cohort of healthy adults and to explore the impact of OFC lesion on the ERP components, especially those reflecting familiarity and recency such as the N2. To our knowledge, this is the first combined lesion-electroencephalography (EEG) study using a Recent Probes task.

## Materials and Methods

### Participants

Sixteen patients with focal lesions located in the OFC and 21 healthy controls were recruited for the study. Participants were right-handed, except one control and one patient who were ambidextrous (Edinburgh handedness inventory, Oldfield, 1971). Healthy controls were recruited by advertisement and personal contact. Patients were recruited through the Department of neurosurgery at Oslo University Hospital and included based on the presence of focal frontal lobe lesion as indicated on pre-existing structural computer tomography (CT) and/or magnetic resonance imaging (MRI) scans. Testing took place at least 2 years after injury or surgery. Participants with a history of serious psychiatric disease, pre-/comorbid neurological disease, premorbid head injury, drug or alcohol abuse requiring treatment, IQ below 85, substantial aphasia, visual neglect, or marked sensory impairment were excluded from participation. The clinical description of the lesions and the evaluation of the controls’ MRI scans were done by a neuroradiologist (P. K. Hol).

After inclusion, two controls and two OFC patients were excluded from the study due to error rates in the Recent Probes task exceeding two standard deviations of their respective group, or due to a highly unbalanced number of errors among conditions (one condition with an error rate at chance level). One additional control participant was removed because of excessive EEG artifacts.

Among the remaining group of 14 OFC patients, 12 had bilateral lesions, one had lesion localized in the right OFC, and one in the left OFC. Twelve patients had lesions caused by a primary extracerebral meningioma brain tumor, and two from traumatic brain injury (TBI) without MRI-based evidence of diffuse axonal injury; see Table 1 for an overview of the patient characteristics.

**Table 1:** Lesion characteristics.

OFC	Etiology	Years since resection post-injury	Lesion size (ccm)			BA Left hemisphere	BA Right hemisphere
			Tot	L	R		
1	Olfactory meningioma	5	42.9	23.1	19.8	10, 11	10, 11
2	TBI	12	24.9	6.4	18.5	11	10, 11, 47
3	TBI	13	157.3	59.7	97.6	8, 9, 10, 11 32, 46, 47, 48	6, 8, 9, 10, 11, 24, 32, 44, 45, 46, 47, 48
4	Olfactory meningioma	11	117.8	56.3	61.5	9, 10, 11, 32, 46, 47	10, 11, 32, 45, 46, 47
5	Olfactory meningioma	5	6.6	3.2	3.4	11	11
6	Olfactory meningioma	7	48.3	11.6	36.7	10, 11	10, 11, 25, 32, 47
7	Olfactory meningioma	11	52.7	26.9	25.8	10, 11, 47	10, 11
8	Olfactory meningioma	11	8.8	1.3	7.5	11	11, 47
9	Olfactory meningioma	12	81.9	45. 7	36.2	10, 11, 32, 45, 46, 47	10, 11, 32, 46, 47
10	Olfactory meningioma	7	3.7	3.7	0	10, 11	-
11	Olfactory meningioma	5	85.7	55.2	30.5	9, 10, 11, 25, 32, 46, 47	10, 11, 47
12	Olfactory meningioma	12	118.7	51	67.7	9, 10, 11, 32, 46, 47	9, 10, 11, 32, 45, 46, 47
13	Olfactory meningioma	2	6.4	0	6.4	-	10, 11
14	Olfactory meningioma	3	32.6	10.1	22.5	11	10, 11, 25

*Etiology, years post-injury, size (total, left, and right hemisphere), and affected Brodmann Areas (BA) for each hemisphere. TBI, Traumatic Brain Injury. The sign “-” was used when no lesion was present in a given hemisphere*.

To equate group sizes, four controls were excluded based on age using the MatchIt R package (Ho et al., 2007). The two remaining groups of 14 participants each did not differ regarding sex, age, years of education, or IQ (estimated based on the Verbal Comprehension and Matrices subtests of the Wechsler Abbreviated Scale of Intelligence (WASI; Wechsler, 1999); see Table 2 and section below “Neuropsychological tests”).

**Table 2:** Subject characteristics per group.

	CTR	OFC	Statistical test
N (% female)	14 (57)	14 (71)	ns.
Age (years)	45.7 (12.7)	48.9 (11.4)	ns.
Education (years)	15.8 (1.5)	15.4 (2.4)	ns.
Total IQ (SD)	110.9 (10.1)	112.6 (8.9)	ns.
Digit Span (SD)	49.00 (7.4)	46.7 (7.9)	ns.
CWIT 1—Color naming (SD)	45.5 (10.0)	50.0 (6.4)	ns.
CWIT 2—Word reading (SD)	44.6 (15.0)	51.4 (8.7)	ns.
CWIT 3—Inhibition (SD)	52.3 (5.8)	55.2 (8.1)	ns.
CWIT 4—Inhibition/switching (SD)	54.0 (6.0)	52.1 (12.0)	ns.

*Comparison of the percentage of female (chi-square test), age, years of education, IQ, working memory (Digit Span) and Inhibition (Color Word Interference Test; CWIT) between the two groups (Student’s t-test for independent samples). The mean value for Total IQ is given in IQ scores, and the mean values for Digit Span Total and Color Word Interference Test in T-scores. The standard deviation is shown in brackets*. ns, not significant.

Patients and controls gave written informed consent to participation in the study. Controls were compensated for participation (600 NOK gift card). The entire research program included a neuropsychological assessment, EEG-recording, as well as structural and functional MRI examination. The study was approved by the Regional Committee for Medical Research Ethics, Region South Norway and was conducted in agreement with the Declaration of Helsinki.

### Lesion Reconstruction

**Figure 1:**
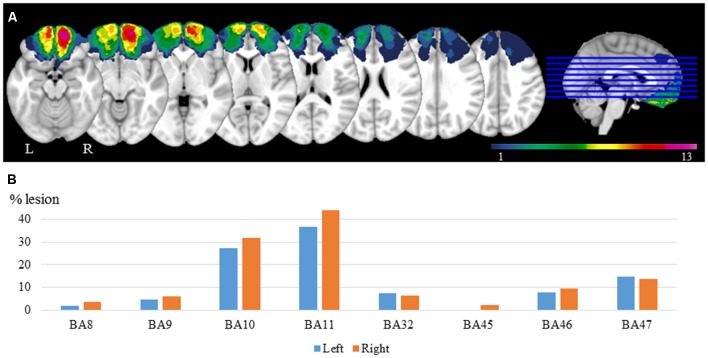
Lesion reconstructions for the orbitofrontal cortex (OFC) group. **(A)** Group overlay. The color code indicates the number of patients with damaged tissue in that area (from 1 to 13). **(B)** Percentage of damaged tissue within each brodmann area (BA) across patients. BAs with less than 2% damage is not presented.

Lesion reconstructions were based on structural MRIs obtained after inclusion and verified by the neurologist and the neurosurgeon in the research group (RK, and TM). Lesions were manually outlined on Fluid Attenuated Inversion Recovery (FLAIR) sequence images (1 × 1 × 1 mm^3^ resolution) for each participant’s brain using MRIcron^2^. High-resolution T1-weighted images were used to help determine the borders of the lesions when required. Each participant’s brain was extracted from the T1 image using the FSL Bet algorithm (FSL^3^) and then normalized to the Montreal Neurological Institute MNI-152 template space using the Statistical Parametric Mapping software (SPM^4^) unified segmentation and normalization procedures, while including the drawn lesions as masks. In addition, the transformation matrix was applied to the individual participant’s FLAIR and lesion mask images. Figure 1 shows the overlay of the individual lesions for the OFC group and the average percentage of damaged tissue within each Brodmann area (BA) across patients.

### Neuropsychological Tests

For all participants, IQ, WM, and inhibition of prepotent verbal responses were measured using Wechsler Abbreviated Scale of Intelligence (WASI; Wechsler, 1999), Digit Span of the Wechsler Adult Intelligence Scale—Third Edition (WASI-III; Wechsler, 1997), and the D-KEFS version of the Color-Word Interference Test (Delis et al., 2001), respectively. Group differences were tested by means of the independent samples Kruskal–Wallis test. Two-tailed Pearson correlation between correct mean RTs for each task condition and each of the neuropsychological test scores was performed.

### Experimental Task

The experimental paradigm used was a Recent-Probes task based on the Sternberg (1966) classical item-recognition task. Participants were seated 80 cm in front of a computer screen. In each trial, participants were presented with a list of five letters displayed on the screen, one at a time, for 500 ms, with an inter-stimulus interval of 500 ms. After a retention period of 4 s they were asked whether a given letter—the probe—was in the list. Participants had 2 s to respond “yes” or “no” by pressing the left or the right button of the response box, respectively. To prevent interference of motor activity due to button presses occurring simultaneously with the electrophysiological signals of interest (supposedly more left-lateralized), all participants were asked to use their left index (“yes” response) and left middle finger (“no” response) to perform the task.

**Figure 2:**
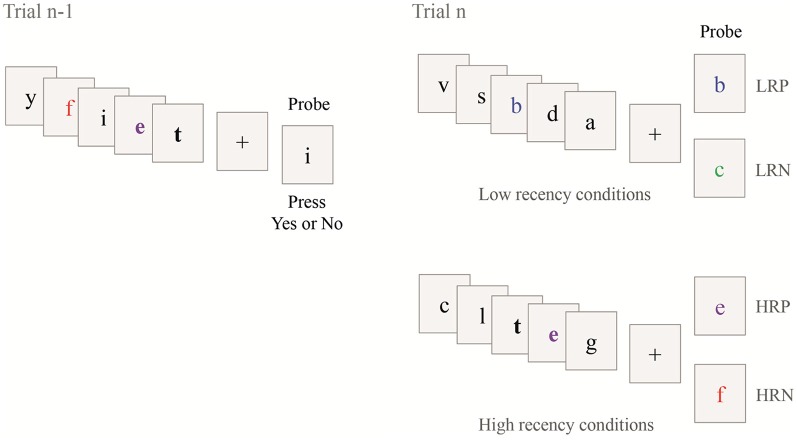
Illustration of the Recent Probes task. Four different conditions composed the task according to the presence or absence of the probe in the current trial (Trial n) and the previous trial (Trial n-1). The two bold letters show the repeated letters in the two consecutive trials. The colors and bold font are used for illustration only. LRP; low recency positive; LRN, low recency negative; HRP, high recency positive, HRP, high recency negative.

The task consisted of four conditions (see Figure 2). The probe letter may be present (positive condition) or absent (negative condition) in the set of letters in the current trial. Further, the probe letter may have occurred in the set of letters in the previous trial (trial n-1, see Figure 2; high recency condition, HR) or not (low recency condition, LR).

For the LR conditions, the probe letter was either presented in the current trial [positive condition (LRP), see the example with the letter “b” in Figure 2] or not [negative condition (LRN), see the example with the letter “c” in Figure 2]. For the HR conditions, the probe was presented in the current and in the previous trial [positive condition (HRP), see the example with the letter “e” in Figure 2] or only presented in the previous trial and not in the current trial [negative condition (HRN), see the example with the letter “f” in Figure 2]. This latter condition is expected to elicit PI. In the two HR conditions, two letters were always shared between the current (trial n) and the previous trial (trial n-1) and one of these two repeated letters was used as probe. No letter was common between two consecutive trials for the two LR conditions. The task included a total of 144 trials, with 36 trials per condition. The trials were presented in three blocks of 10 min each, consisting of 48 trials randomly sorted between conditions (except for the first trial of each block which was assigned as an LR trial). Time on task was 30 min. Short breaks between blocks resulted in a total task duration of approximately 35 min.

### Behavioral Analysis

The RT to the probe was recorded for each trial. Trials with RT faster than 400 ms^5^ or exceeding three standard deviations of the average RT of any given participant were excluded. The remaining trials were included in subsequent analyses of response latency (based on the mean RT of correct trials per subject) and error rate (based on the percentage of incorrect trials). For both analyses, we ran repeated measures ANOVAs using group (controls vs. OFC patients) as between-subjects factor, and recency (HR vs. LR trials), and response (negative vs. positive answer) as within-subject factors. The analysis of homogeneity of variance between groups was analyzed using Levene’s test. The age variable for both groups, as well as lesion volume and years since tumor resection or injury for the OFC group, were added as covariates. The analyses were conducted using R software (RC Team, 2015).

Finally, a *post hoc* analysis was conducted to examine the PI effect by subtracting the mean RTs, as well as the mean percentage of errors, of LRN from HRN conditions for each subject. We then performed one-way ANOVAs with this new variable (using an alpha level of *p* < 0.05).

### EEG Acquisition

Participants were seated in a Faraday-shielded room 80 cm from an LCD monitor with a 60 Hz refresh rate. EEG was recorded at a 1,024 Hz sampling rate using a 64-channel Biosemi Active Two system^6^ with electrodes placed using the Biosemi headcap in accordance with the International 10-20 system. Two vertical electrooculography (EOG) electrodes were placed above and below the right eye and two horizontal EOG electrodes were placed at the participants’ left and right canthi. Two reference electrodes for later offline re-referencing were also placed on the left and right earlobes.

### EEG Preprocessing

Continuous EEG data were filtered offline with a 0.01–130 Hz bandpass filter with a notch filter at 50 Hz. The data were re-referenced to a common average reference (with the exclusion of the six external electrodes) after removal and interpolation of bad channels using an automatic detection with a limit of activity probability set at 5 standard deviations. Epochs of 14 s (11 s before- and 3 s after probe presentation) were first created. Following the recommendation of Delorme et al. (2007), bad epochs were detected and removed using independent component analysis (ICA). A first ICA was computed to reject ICA epochs with extremely large potential fluctuations (artifacts above 1,000 μV) and then iteratively rejecting ICA epochs based on a threshold of 5 standard deviations. A second ICA decomposition was computed on the remaining EEG epochs to remove ICA components containing eye-movements, blinks, and muscle artifacts. Clean EEG epochs were then segmented according to six conditions for each participant. The two first conditions were created according to recency only: HR, LR and were used for the analysis of the encoding and maintenance phases, as the probe content is necessarily unknown during those phases. The other four conditions were based on both recency and response: LRN, LRP, HRN, and HRP, and were created for the analyses of the probe phase. Only correct trials were retained for EEG analysis. Within each condition, the electrophysiological signal was demeaned and detrended, and a low pass filter (20 Hz) was applied to each trial. Within each group of participants (controls or OFC patients), a grand average across trials and participants of any given condition was then computed. Preprocessing routines were performed using EEGLAB (Delorme and Makeig, 2004) and FieldTrip (Oostenveld et al., 2010) toolboxes in MATLAB (MathWorks Inc., Natick, MA, USA).

### ERP Analysis

We divided the entire trial (from 11 s before- to 3 s after probe presentation) into three time windows of interest: the encoding of the five successively presented letters (from 9 to 4 s before the probe presentation), the maintenance of the letters (from 4 s leading up to the probe presentation), and the probe period (500 ms pre- to 2 s post- probe onset).

We employed a similar approach to the one used by Ye et al. (2017), consisting of finding the significant spatiotemporal differences between conditions within each group first and then performing between-group comparisons on that outcome. Within each group, we compared HR and LR conditions during the encoding and maintenance phases, using a cluster-based permutation *t*-test. We then detected if there were ERP differences between the four conditions (HRN, HRP, LRN, LRP) during the probe presentation periods, using a cluster-based permutation *F*-test. To define the spatiotemporal cluster of interest, we used permutation statistics within each group of participants, as implemented in Fieldtrip (Monte Carlo method; 1,000 iterations; *p* < 0.05). To correct for multiple comparisons, a cluster approach was used (Maris and Oostenveld, 2007). Clusters statistics were obtained by summing the *t*-values (for *t*-test) or *F*-values (for *F*-test) that were adjacent in space and time for which the alpha level was below 0.05. The minimum number of neighboring electrodes required to form a cluster was three. The cluster *p*-value was then obtained by comparing the cluster statistic to a null distribution statistic obtained by randomly switching condition labels within participants’ trials 1,000 times. The clusters were considered as independently significant when the sum of *F*-values exceeded 95% of the null distribution.

For each spatiotemporal cluster showing a significant difference between conditions, the mean amplitude of the respective time window and electrodes were extracted and averaged for each condition. Using these means, a mixed repeated-measures ANOVA was performed with group (controls vs. OFC patients) as a between-subjects factor, and recency (HR vs. LR trials) and response (negative vs. positive answer) as within-subject factors. Finally, an ANCOVA was conducted by adding the behavioral performance as a covariate. These analyses were computed using IBM SPSS Statistics 25.0.

## Results

### Behavioral Results

**Figure 3:**
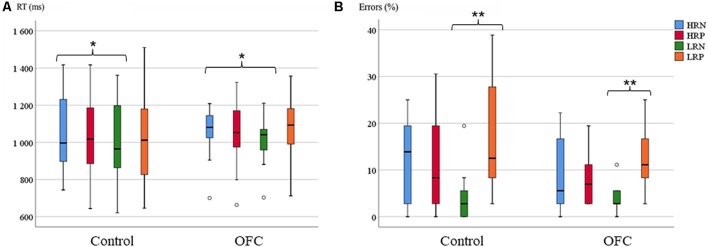
Response latency and accuracy on the Recent Probes task. **(A)** Boxplots of the mean reaction time (RT; in ms) per group and condition. **(B)** Boxplots of the mean percentage of errors per group and condition (right panel). The horizontal line in each box represents the median, and the bars extending vertically from the boxes (and the circles) indicate the variability outside the upper and lower quartiles. Asterisks indicate the significant differences (*p ≤ 0.05, **p ≤ 0.005).

The repeated measures ANOVA performed on the RT of correct trials revealed no group difference in response latency (mean for the control group: 1,020 ms, SD: 54; mean for OFC group: 1,046 ms, SD: 38). At the condition level, we found a main effect of recency (*F*_(1,26)_ = 5.63, *p* < 0.05, *r* = 0.42), reflecting longer RTs for high- compared to low recency probes, but no interaction with group. Whereas the response effect was not significant, the interaction between response and recency was significant (*F*_(1,26)_ = 6.39, *p* < 0.05, *r* = 0.44). Overall, the RTs for the HRN condition was slower than the other conditions, especially the LRN condition, documenting the existence of PI (RTs mean for HRN: 1,064 ms, HRP: 1,056 ms, LRN: 1,020 ms, LRP: 1,058 ms, Figure 3A). However, the direct comparison of PI (the difference in RTs between the HRN and the LRN conditions) between groups revealed no significant effect, but the analysis of homogeneity of variances showed that RTs in the HRN condition tended to be more variable for the control group (*SD* = 52) than the OFC group (*SD* = 35; *F*_(1,26)_ = 3.9, *p* = 0.058, *r* = 0.36)^7^.

The analysis of response accuracy showed no significant group effect (11% errors for controls and 8% for OFC patients). A significant main effect of response type was found (*F*_(1,26)_ = 10.34, *p* < 0.005, *r* = 0.53), but no group interaction. There was no significant main effect of recency, but an interaction between recency and response was significant (*F*_(1,26)_ = 39.99, *p* < 0.001, *r* = 0.78). Overall, fewer errors were made in the LRN condition compared to the other conditions (HRN: 11%, HRP: 10%, LRN: 4%, LRP: 14%, Figure 3B). The *post hoc* analysis of the PI effect over the percentage of errors was not significantly different between groups. The analysis of homogeneity of variances between groups revealed significant group difference for all the conditions except for LRN (HRN (*SD* = 2.5 for control and *SD* = 2 for OFC): *F*_(1,26)_ = 4.28, *p* < 0.05, *r* = 0.37, HRP (*SD* = 2.5 for control and *SD* = 1 for OFC): *F*_(1,26)_ = 4.38, *p* < 0.05, *r* = 0.38, LRP (*SD* = 3 for control and *SD* = 1.5 for OFC): *F*_(1,26)_ = 6.16, *p* < 0.05, *r* = 0.44). The error rates were more variable within the control group than within the OFC group.

No effect of age was reported significant between-group and within conditions for the latency or the error rate. Within the OFC group, no significant effect of the lesion volume nor effect of years after resection was found impacting the behavioral results.

None of the neuropsychological measures significantly correlated with the behavioral results of the Recent Probes task. The groups were not significantly different in estimated IQ or on any of the neuropsychological tests (Digit Span forward and backward, and Color-Word Interference test). The total lesion volume of OFC patients did not correlate with any neuropsychological test performance measured in this study.

### Electrophysiological Results

#### The Probe Phase

The permutation *F*-test between the four conditions during the probe period was conducted separately for each group of participants over the time-course for each electrode. The results revealed one extensive cluster that showed significant conditions difference for the control group (*p* < 0.001) from 329 ms to 479 ms after probe onset. The spatial distribution, as well as the ERP time-course of the electrodes constituting the cluster, allowed division into two distinct groups of electrodes. The first with a right hemisphere Centro-posterior distribution (referred to as cluster 1) and the second with a left-lateralized frontal distribution (referred to as cluster 2). Surprisingly, no late cluster was found in the control group, i.e., no condition effect over late ERPs, such as the LPC, were observed. To verify if this lack of ERP modulation could be explained by the detrending algorithm used in preprocessing, a similar analysis was performed without this step. The results showed no other significant difference than the two previously described.

**Figure 4:**
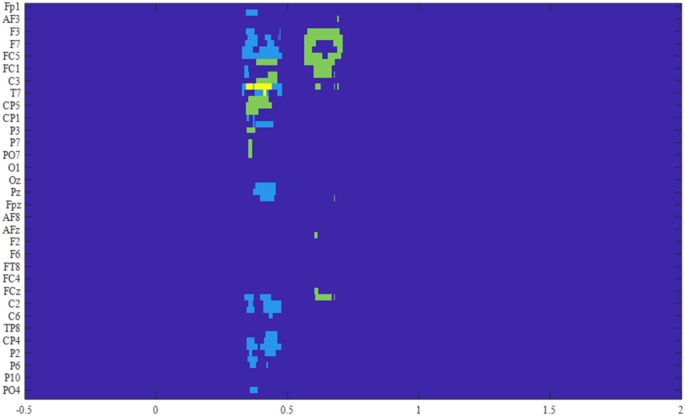
Results of the *F*-tests across the four conditions for the two groups. The significant electrodes within the time window of interest are represented for each cluster, in light blue for the control group, in green for the OFC group, and in yellow for the overlap between groups.

For the OFC group analysis, two clusters revealed a significant difference between conditions. The first one from 565 ms to 710 ms (*p* < 0.005, referred to as cluster 3) had a similar left frontal distribution as cluster 2, and a second one from 345 ms to 463 ms (*p* < 0.01, referred to as cluster 4) occurred within the time window of the two clusters observed for the control group (Figure 4).

Among these four clusters, the behavioral performance did not significantly covary with ERP amplitudes in any of the task conditions.

### Significant Electrode Clusters for the Control Group

#### Time-Course of Cluster 1

**Figure 5:**
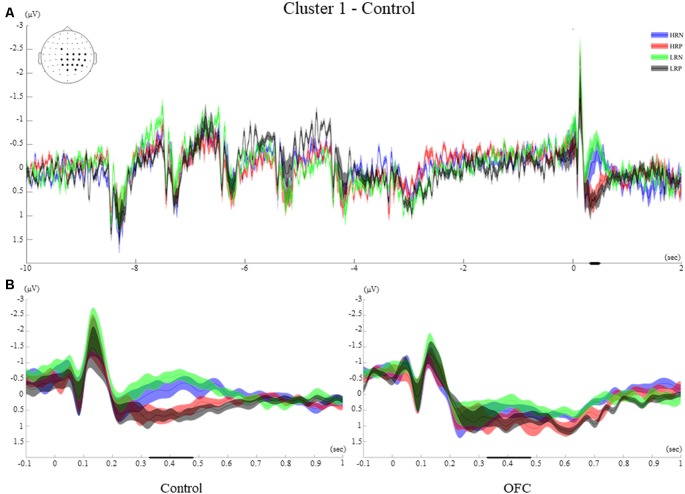
Cluster 1. **(A)** Grand average event-related potentials (ERPs) of electrodes included in cluster 1 of the control group. The electrodes included in each cluster are highlighted in the scalp maps. The grand average ERPs of the four conditions are illustrated for the entire duration of the trial (12 s). The 0 on the x-axis represents the probe presentation. The bold lines on the x-axis illustrate the time points with significant *F*-values. The negative polarity is presented upward. **(B)** Comparison of the grand average ERPs of the four conditions between the control group and the OFC group for cluster 1. The bold line on the x-axis represents the time window within which the amplitude was extracted and used in the repeated measures ANOVA tests. The negative polarity is presented upward. The shaded area around the ERP represents the standard error of the mean (SEM).

Cluster 1 involved 18 Centro-posterior electrodes, mainly lateralized to the right hemisphere and the central midline (FC1, C1, CP1, P1, POz, Pz, CPz, Cz, C2, C4, C6, CP6, CP4, CP2, P2, P4, P6, PO4, Figure 5A). The time-course of this cluster after the probe presentation first revealed early visual ERPs (P1, N1) which were followed by a positive ERP peaking at 220 ms in all four conditions. During the time window of significant condition differences (329 ms–479 ms post probe onset), the ERPs of the LRN and HRN conditions returned to baseline, while the LRP and HRP conditions showed a sustained positive-going activity following the peak P3 that lasted until around 600 ms after probe presentation.

#### Between-Group Effects of Cluster 1

The *post hoc* ANOVA between groups over the mean amplitude across the time window and electrodes of cluster 1 showed a significant main effect of the response (*F*_(1,26)_ = 33.43, *p* < 0.001, *r* = 0.75), that was modified by an interaction with group (*F*_(1,26)_ = 5.97, *p* < 0.05, *r* = 0.43). These results are consistent with the observation that, in the control group only, the two positive response conditions elicited more positive-going ERP amplitudes than the negative response conditions. For the OFC group, the four conditions elicited a sustained positivity onsetting at 220 ms after the probe that was not significantly modulated by response type (Figure 5B).

#### Time-Course of Cluster 2

**Figure 6:**
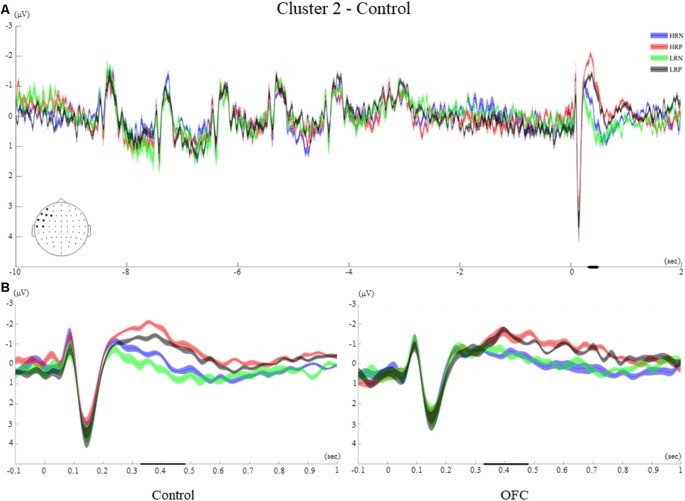
Cluster 2. **(A)** Grand average ERPs of electrodes included in cluster 2 of the control group. The electrodes included in each cluster are highlighted in the scalp maps. **(B)** Comparison of the grand average ERPs of the four conditions between the control group and the OFC group for cluster 2. The bold line on the x-axis represents the time window within which the amplitude was extracted and used in the repeated measures ANOVA tests. The shaded area around the ERP represents the SEM.

Cluster 2 included eight left frontal electrodes (AF7, F3, F5, F7, FT7, FC5, C5, T7, Figure 6A). The time-course of this frontal cluster after probe presentation showed visual ERPs that were temporally inverted relative to cluster 1 (N1, P1), followed by an ERP of negative polarity which was different across conditions. A small negativity peaking around 220 ms was observed for the two negative response conditions, while an enhanced negativity peaking around 400 ms was found for the positive response conditions.

#### Between-Group Effects of Cluster 2

The between-groups analysis showed a significant main effect of response type (*F*_(1,26)_ = 33.43, *p* < 0.001, *r* = 0.70), but no significant interaction with the group. During the time window of interest, the two positive response conditions elicited a larger and later negative polarity ERP compared to the two negative response conditions for both groups of participants. A significant main effect of recency (*F*_(1,26)_ = 6.6, *p* < 0.05, *r* = 0.45) was modified by an interaction with group (*F*_(1,26)_ = 5.76, *p* < 0.05, *r* = 0.42). Indeed, the recency manipulation evoked significantly different amplitudes within the control group only, with more negative amplitudes for the high recency (HRN and HRP) than for the low recency (LRN and LRP) conditions (Figure 6B).

### Significant Electrode Clusters for the OFC Group

#### Time Course of Cluster 3

**Figure 7:**
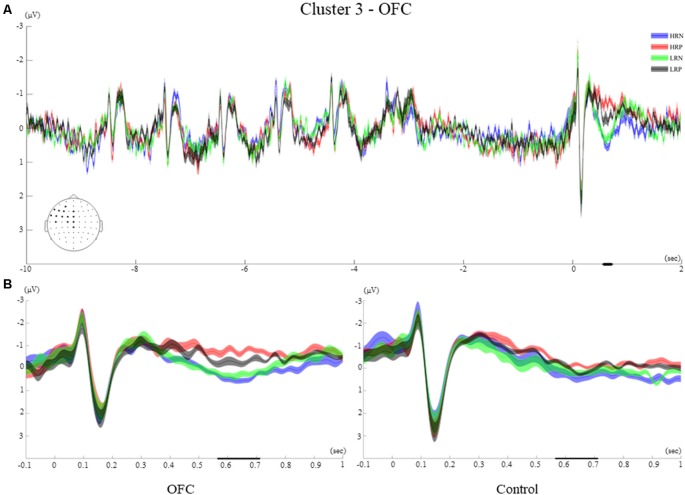
Cluster 3. **(A)** Grand average ERPs of electrodes included in cluster 3 of the OFC group. The electrodes included in each cluster are highlighted in the scalp maps. **(B)** Comparison of the grand average ERPs of the four conditions between the control group and the OFC group for cluster 3. The bold line on the x-axis represents the time window within which the amplitude was extracted and used in the repeated measures ANOVA tests. The shaded area around the ERP represents the SEM.

Cluster 3 showed a late effect, from 565 ms to 710 ms and included left frontocentral and midline electrodes (AF3, F3, F5, F7, FT7, FC5, FC3, FC1, C1, C5, Pz, Fz, FCz, Cz, Figure 7A). The time-course of this cluster was similar to that of cluster 2 of the control group with visual ERPs (N1, P1), followed by an ERP of negative polarity peaking around 300 ms. The significant difference between conditions was observed when a positive polarity ERP at 630 ms appeared for the negative response conditions only.

#### Between-Group Effects of Cluster 3

The repeated measures ANOVA comprising the recency and response factors revealed a significant main effect of the response (*F*_(1,26)_ = 24.5, *p* < 0.001, *r* = 0.70), and a response by group interaction (*F*_(1,26)_ = 5.32, *p* < 0.05, *r* = 0.41). These results are consistent with the observation that the two negative response conditions elicited a later and more positive-going ERP amplitude for the OFC group only (Figure 7B). Moreover, a significant interaction between the recency and response factors (*F*_(1,26)_ = 6.33, *p* < 0.05, *r* = 0.44), reflected that the biggest difference was between HRN and LRP conditions across groups.

#### Time-Course of Cluster 4

**Figure 8:**
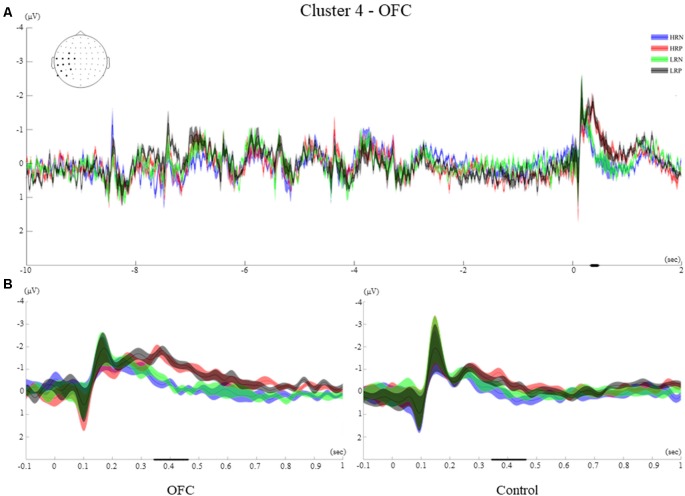
Cluster 4. **(A)** Grand average ERPs of electrodes included in cluster 4 of the OFC group. The electrodes included in each cluster are highlighted in the scalp maps. **(B)** Comparison of the grand average ERPs of the four conditions between the control group and the OFC group for cluster 4. The bold line on the x-axis represents the time window within which the amplitude was extracted and used in the repeated measures ANOVA tests. The shaded area around the ERP represents the SEM.

The time-course of showed first visual ERPs (P1, N1), followed by an ERP of negative polarity peaking at 260 ms. The significant effect is observed during a similar time window as the clusters found for the control group, from 345 ms to 463 ms. A larger negative polarity ERP is observed for the two positive conditions compared to negative conditions. This effect was evident over the left hemisphere, mainly over the Centro-posterior electrodes (FC3, C1, C3, C5, T7, TP7, CP5, CP3, P3, P7, P9, PO7, Figure 8A).

#### Between-Group Effects of Cluster 4

The between-groups analysis revealed a significant main effect of the response (*F*_(1,26)_ = 30.5, *p* < 0.001, *r* = 0.73), as well as an interaction of response and group (*F*_(1,26)_ = 4.76, *p* < 0.05, *r* = 0.39). The negative polarity ERPs observed at around 250 ms remained sustained for the two positive response conditions while the two negative response conditions returned to baseline for the OFC group. Despite a similar time-course for the control group, no sustained activity was observed for this cluster (Figure 8B).

### The Encoding and Maintenance Phases

The *t*-test performed over the letter encoding and maintenance periods did not show significant ERP differences between the HR and LR conditions for any of the groups of participants. The ERPs elicited by the letters presented on the screen was not modulated by the recency manipulation. Moreover, the recency effect did not impact the way letters were retained in WM during the delay interval (see examples of ERPs elicited during the encoding and maintenance for the two groups in Figures 5–8A of the four clusters).

## Discussion

This study examined the role of the OFC in WM using a Recent Probes task. Unexpectedly, the behavioral analyses suggest that patients with OFC lesion performed the task properly despite their brain damage. Although OFC electrophysiological responses differed spatiotemporally from the control group, the maintenance of letters in WM, the evaluation of the context of the probe, and the decision to accept or reject a probed letter were preserved in our patient cohort. Moreover, the sensitivity to the recency of the probe was preserved at the behavioral level despite the differential modulation of ERP markers in the OFC group.

### Behavioral Findings

The two groups showed similar behavioral patterns across task conditions. For both groups, the response time to the probe letter was slower when the probe had been present in the memory set of the previous trial (i.e., the recency effect), especially if the probe was absent in the current one (HRN condition; Monsell, 1978; Jonides et al., 1998), reflecting the PI effect. Moreover, this PI was not different between groups. The weaker recency effect compared to that usually observed in earlier studies, can be partly explained by the wider age range in our study (Jonides et al., 1998; Du et al., 2008; Yi and Friedman, 2014) as controls were selected to match the age range of the OFC cohort. Indeed, it has been shown that performance on this type of WM tasks changes over life (Jonides et al., 2000; Thompson-Schill et al., 2002; Park and Reuter-Lorenz, 2009) and may relate to a reduction in inhibition or in attention (Tays et al., 2008; Yi and Friedman, 2014). Regarding the error rate, the OFC patients were as accurate in their judgments as the controls. Overall, the number of errors was smaller for the negative response conditions compared to the positive conditions, indicating that the rejection of probes was cognitively less demanding. Notably, accuracy was enhanced when the rejected probe had not been presented in the previous trial.

More variability in response latency and accuracy were observed within the control group than the OFC group. One explanation could come from a potentially wider choice of strategies that healthy participants could apply to perform the task (for instance, keeping in mind and rehearsing the five letters or creating pseudo-words (Braver et al., 2007) compared to patients with a lesion in the OFC. However, participants were not asked about their choice of strategy.

Standard neuropsychological tests did not reveal any deficits on measures of general intellectual ability, selective attention, processing speed, memory span and WM (Digit Span forward and backward, respectively) for this patient cohort. This is in line with other studies reporting no general deficit in attention or WM after OFC damage (Müller et al., 2002; Barbey et al., 2011). Moreover, IQ and Digit Span were not correlated with performance on the Recent Probes task for any group.

### Electrophysiological Findings

Examination of the electrophysiological time-courses, spanning the three different phases composing the Recent Probes task, showed that in both groups the encoding and the maintaining of letters in WM were similar for the HR and LR conditions. This is in line with the findings of other studies (D’Esposito et al., 1999b; Badre and Wagner, 2005; Braver et al., 2007). However, the analysis of the recognition probe period revealed spatiotemporal differences in ERPs between the four task conditions. This suggests that whether a probe was present or not in the previous trial did not impact the encoding and the maintaining of the subsequent trial information, i.e., the task condition was only determined by the probe.

While the ERP condition differences observed for the control group occurred within the same time window and involved bilateral electrodes, the differences for the OFC group were left-lateralized and occurred during two consecutive time windows. The first cluster observed for the OFC group (cluster 3) shared common left electrodes with cluster 2 of the control group but occurred later in time, while the second cluster of the OFC group (cluster 4) occurred at the same timing as the control group but involved a different set of left posterior electrodes.

The electrophysiological results did not concur with behavioral observations. Indeed, task condition effects observed at the ERP level were mostly driven by response manipulation. The probe recency effect only impacted ERPs for the left frontal electrodes in cluster 2 of the control group. This observation is in line with previous work by Milner et al. (1991) showing that the left mid-lateral frontal cortex plays a crucial role in performing recency judgments for verbal information. Interestingly, no recency effect on ERPs was observed for the OFC group. In our study, the subtle amplitude modulation of the negative-going ERP component elicited by the recency of the probe may be due to the observation that the response effect was equivalent to, if not stronger than, the recency effect.

The electrophysiological results observed for the control group during the time window of interest were comparable with those reported in studies using similar experimental designs. Our experimental manipulation elicited two main ERP components peaking during the same time window (between 200 and 300 ms after probe onset), but with different spatial distributions: a left frontal negative-going component and a right centroparietal positive component (Du et al., 2008; Tays et al., 2008, 2009; Zhang et al., 2010; Yi and Friedman, 2014). We found that the two components were sensitive to the “yes” and “no” responses, but only the frontal negativity (cluster 2) was sensitive to the recency-manipulation. These results are in accordance with Du et al. (2008) and Folstein and Van Petten (2008), who reported a modulation of a N2 and a P3 by the positive and negative responses, but also a modulation of the N2 by the response-conflict and inhibitory processes induced by PI (Tays et al., 2008, 2009). However, contrary to Du et al. (2008); we observed a larger and more frontal N2 as well as an earlier and larger P3 for the positive responses. These ERP differences may be due to task design differences and the cognitive processes engaged. For example, Du et al. (2008) directed forgetting of some letters, while our study required maintenance of the entire set of letters until the probe appeared. This explicit manipulation of items kept in WM might lead to different modulation of the brain network involved in PI. However, we failed to reproduce the recency modulation observed by Zhang et al. (2010) as no modulation of the amplitude of the LPC was observed for the controls. These results indicate that recency manipulation influences the brain activity of healthy participants only shortly after the probe presentation (around 200 ms).

In our study, the N2 observed in the OFC group had a more posterior distribution than the control group. The N2 was sensitive to the response manipulation but not to the recency effect. The LPC was also modulated by the response only. These results indicate that neural markers of the brain network engaged in the “yes” or “no” response to the probe were preserved in the OFC group despite being spatiotemporally shifted.

The finding that none of the ERP components identified for the OFC group were modulated according to probe recency, while the behavioral parameters were influenced, was surprising. One may speculate that this apparent mismatch in our results is related to the fact that these effects are small in magnitude or susceptible to interindividual variability within the OFC group.

Although the cognitive strategies involved during task performance might have been different, the resulting overall behavioral performance was similar across groups. One explanation for the preserved task performance could be that, despite being part of the network subserving proactive cognitive control (Badre and Wagner, 2005; Nee et al., 2007; Irlbacher et al., 2014), the OFC is not critical for the resolution of PI. Preservation of brain regions such as the VLPFC and the DLPFC may be sufficient to maintain adequate task performance (Jonides et al., 1998; Postle et al., 2004; Wager et al., 2005; Jonides and Nee, 2006; Burgess and Braver, 2010; Barbey et al., 2013).

Another explanation could be the reorganization of function compensating for OFC damage. In our study, we observed that the clusters of significant difference were both left-lateralized for the OFC group while the control group presented a right and a left-lateralized cluster. Moreover, the OFC group showed condition difference in two distinct time windows. These spatiotemporal differences could reflect compensatory mechanisms. Voytek and Knight (2011) suggested that patients suffering from slow-growing brain lesions (as most of the patients in our cohort) have more efficient compensatory mechanisms compared to patients with acute brain damage. Moreover, the authors proposed that deficits caused by damage in frontal regions (compared to posterior regions) are more likely to recover due to more distributed brain networks supporting function, thus being more resistant to focal brain damage (see also Anderson, 2007). We acknowledge that a significant correlation between behavioral performance and ERPs would have strengthened this interpretation. However, the correlation between ERPs and behavioral parameters tends to be weak, likely because the two sets of measures provide different windows into brain function. Small sample sizes, as in the current study, is also a limitation. Group differences in physiological- but not in behavioral data can, therefore, provide insights about the information processing in a system that, if gleaned from behavioral data only, appears to be normal. The altered ERPs may represent a processing deficit that behavioral measures are not sensitive enough to detect. An alternative interpretation may be a difference in task strategy between the groups, but this remains speculative and the overall pattern of behavior does not point to different strategies.

## Conclusion

The OFC group had a similar sensitivity to the experimental conditions of a Recent Probes task and performed at the same high accuracy as healthy controls. However, the electrophysiological data indicate that the two groups differed in the modulation of the brain networks supporting task performance. The findings suggest that neural reorganization compensates for OFC damage.

## Data Availability Statement

The datasets generated for this study are available on request to the corresponding author.

## Ethics Statement

The studies involving human participants were reviewed and approved by Regional Committee for Medical Research Ethics, Region South Norway. The patients/participants provided their written informed consent to participate in this study.

## Author Contributions

TE, JL, and AL contributed to the conception and design of the study. TM, A-KS, and RK helped with the recruitment and clinical characterization of patients. IF, AB, MF, SL, and AL helped to collect the data and worked on the lesion reconstruction together with TM and A-KS. AL, RH, and IF performed the statistical analysis. RK, A-KS, and TE provided substantial contributions to the interpretation of the data. AL wrote the first draft of the manuscript. All authors contributed to manuscript revision and improvement, read and approved the submitted version.

## Conflict of Interest

The authors declare that the research was conducted in the absence of any commercial or financial relationships that could be construed as a potential conflict of interest.

## Footnotes

^1^The orbitofrontal cortex, commonly referred to as the ventromedial part of the prefrontal cortex or the frontopolar prefrontal cortex in the literature, will only be referred to as OFC in the remaining of this article.

^2^www.mccauslandcenter.sc.edu/mricro/mricron/

^3^https://fsl.fmrib.ox.ac.uk/fsl/fslwiki/FSL

^4^www.fil.ion.ucl.ac.uk/spm/

^5^This latency was suggested as the zero intercept in this type of task by Sternberg (1966). In our study, the percentage of correct trials with RT faster than 400 ms was 0.2%.

^6^http://www.biosemi.com/

^7^The effect of homogeneity of variances between groups was weakened by the presence of an unusually fast OFC patient who was the only one in our pool of patients suffering traumatic brain injury early in life (14 years old). A possible explanation for this participant’s speed may be attributed to brain plasticity, which could have enhanced recovery of functions (for review see Johnston, 2004).
